# 3-(4-Hydroxy­phenyl­imino)indolin-2-one

**DOI:** 10.1107/S1600536810012754

**Published:** 2010-04-10

**Authors:** Yan Meng

**Affiliations:** a School of Environmental Engineering, Chang’an University, South Second Cycle Road 368, Xi’an 710054, Shannxi, People’s Republic of China

## Abstract

In the title compound, C_14_H_10_N_2_O_2_, the dihedral angle between the indole and benzene rings is 61.63 (4)°. In the crystal structure, centrosymmetrically related mol­ecules are linked into dimers by N—H⋯O hydrogen bonds, generating rings of graph-set motif *R*_2_^2^(8). The dimers are further connected into a three-dimensional network by O—H⋯O and C—H⋯O hydrogen bonds.

## Related literature

For the synthesis and applications of 3-imino­indole-2-one derivatives, see: Chen, Tang, Zhou & Hao (2009 ▶); Chen, Tang, Wang *et al.* (2009 ▶); Chen *et al.* (2007 ▶); Liu *et al.* (2003 ▶).
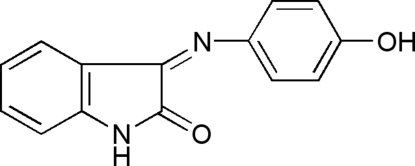


## Experimental

### 

#### Crystal data

C_14_H_10_N_2_O_2_
*M* *_r_* = 238.24
Monoclinic, 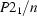

*a* = 5.7662 (17) Å
*b* = 15.383 (5) Å
*c* = 12.898 (4) Å
β = 100.479 (16)°
*V* = 1124.9 (6) Å^3^
*Z* = 4
Mo *K*α radiation
μ = 0.10 mm^−1^
*T* = 296 K
0.36 × 0.27 × 0.21 mm

#### Data collection

Bruker SMART CCD diffractometer
Absorption correction: multi-scan (*SADABS*; Bruker, 2002 ▶) *T*_min_ = 0.889, *T*_max_ = 0.927
6932 measured reflections
2795 independent reflections
1878 reflections with *I* > 2σ(*I*)
*R*_int_ = 0.031

#### Refinement

*R*[*F* ^2^ > 2σ(*F* ^2^)] = 0.046
*wR*(*F* ^2^) = 0.125
*S* = 1.03
2795 reflections
163 parameters
H-atom parameters constrained
Δρ_max_ = 0.14 e Å^−3^
Δρ_min_ = −0.21 e Å^−3^

### 

Data collection: *SMART* (Bruker, 2002 ▶); cell refinement: *SAINT-Plus* (Bruker, 2002 ▶); data reduction: *SAINT-Plus*; program(s) used to solve structure: *SHELXS97* (Sheldrick, 2008 ▶); program(s) used to refine structure: *SHELXL97* (Sheldrick, 2008 ▶); molecular graphics: *SHELXTL* (Sheldrick, 2008 ▶); software used to prepare material for publication: *SHELXTL*.

## Supplementary Material

Crystal structure: contains datablocks I, global. DOI: 10.1107/S1600536810012754/rz2431sup1.cif

Structure factors: contains datablocks I. DOI: 10.1107/S1600536810012754/rz2431Isup2.hkl

Additional supplementary materials:  crystallographic information; 3D view; checkCIF report

## Figures and Tables

**Table 1:** Hydrogen-bond geometry (Å, °)

*D*—H⋯*A*	*D*—H	H⋯*A*	*D*⋯*A*	*D*—H⋯*A*
O2—H2*B*⋯O1^i^	0.82	1.96	2.7628 (17)	165
N1—H1*A*⋯O1^ii^	0.86	2.12	2.9071 (16)	153
C10—H10*A*⋯O2^iii^	0.93	2.38	3.275 (2)	160
C11—H11*A*⋯O1^i^	0.93	2.52	3.117 (2)	122

Symmetry codes: (i) 
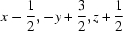
; (ii) 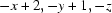; (iii) 
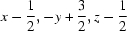
.

**Fig. 1.**
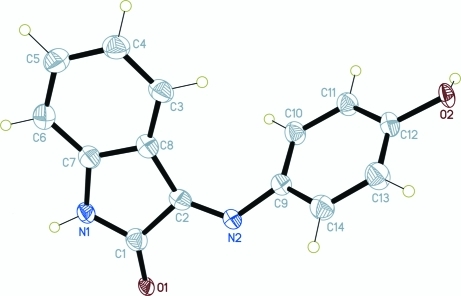
The molecular structure of the title compound, with the atom-numbering scheme and 30% probability displacement ellipsoids.

**Fig. 2.**
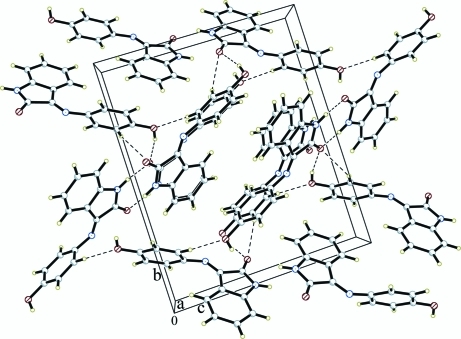
Packing of the title compound viewed along the a axis. Intermolecular hyderogen bonds are drawn as dashed lines.

## supplementary crystallographic information

### Comment

3-Imine-indole-2-one derivatives have driven much attentions for their bioactivities such as anti-bacterial, anti-virus and neuroprotective activities (Chen, Tang, Zhou & Hao, 2009; Chen, Tang, Wang  *et al.*, 2009; Chen *et al.*, 2007; Liu *et al.*, 2003). The title compound, 3-(4-hydroxyphenylimino)indolin-2-one, has been synthesized by the condensation reaction of isatin and 4-aminophenol, and its crystal structure is reported herein.

The X-ray structural analysis confirmed the assignment of the structure from spectroscopic data. The molecular structure is depicted in Fig. 1, and a packing diagram of is depicted in Fig. 2. Geometric parameters of the title compound are in the usual ranges. The dihedral angle between the indole and benzene rings is 61.63 (4)°. The C2–N2–C9 angle is 122.97 (12)°, and the C8–C2–N2–C9 torsion angle is -9.0 (3). In the crystal structure, centrosymmetrically related molecules are linked into dimers by N—H···O hydrogen bonds (Table 1) generating rings of graph set motif *R*^2^_2_(8). The dimers are further connected into a three-dimensional network by O—H···O and C—H···O hydrogen bonds.

### Experimental

Isatin (1 mmol) was dissolved in methanol (20 ml) and a methanol solution of 1.2 mmol 4-aminophenol (10 ml) was added dropwise, until the disappearance of isatin, as evidenced by thin-layer chromatography. The solvent was removed in vacuo and the residue was separated by column chromatography (silica gel, petroleum ether/ethyl acetate = 1:1 *v*/*v*), to give the title compound. Yellow single crystals of the title compound suitable for X-ray analysis were obtained on slow evaporation of a methanol solution (30 ml) of the title compound (30 mg) over a period of 4 d. ^1^H-NMR (D_6_—DMSO, 400 MHz): 10.92 (1*H*, s), 9.56 (1*H*, s), 7.32 (2*H*, m), 6.86 (4*H*, m), 6.74 (3*H*, m); MS (EI) m/z: 238 (M^+^).

### Refinement

All H atoms were placed at calculated positions and refined as riding, with C—H = 0.93 Å, N—H = 0.86 Å and O—H = 0.82 Å, and with *U*_iso_(H) = 1.2*U*_eq_(C, N) or 1.5*U*_eq_(O).

### Crystal data

**Table:**

C_14_H_10_N_2_O_2_	*F*(000) = 496.0
*M**_r_* = 238.24	*D*_x_ = 1.407 Mg m^−^^3^
Monoclinic, *P*2_1_/*n*	Mo *K*α radiation, λ = 0.71073 Å
Hall symbol: -P 2yn	Cell parameters from 7285 reflections
*a* = 5.7662 (17) Å	θ = 1.5–25.0°
*b* = 15.383 (5) Å	µ = 0.10 mm^−^^1^
*c* = 12.898 (4) Å	*T* = 296 K
β = 100.479 (16)°	Block, colourless
*V* = 1124.9 (6) Å^3^	0.36 × 0.27 × 0.21 mm
*Z* = 4	

### Data collection

**Table:**

Bruker SMART CCD diffractometer	2795 independent reflections
Radiation source: fine-focus sealed tube	1878 reflections with *I* > 2σ(*I*)
graphite	*R*_int_ = 0.031
φ and ω scans	θ_max_ = 29.0°, θ_min_ = 2.1°
Absorption correction: multi-scan (*SADABS*; Bruker, 2002)	*h* = −7→7
*T*_min_ = 0.889, *T*_max_ = 0.927	*k* = −14→20
6932 measured reflections	*l* = −17→16

### Refinement

**Table:**

Refinement on *F*^2^	Primary atom site location: structure-invariant direct methods
Least-squares matrix: full	Secondary atom site location: difference Fourier map
*R*[*F*^2^ > 2σ(*F*^2^)] = 0.046	Hydrogen site location: inferred from neighbouring sites
*wR*(*F*^2^) = 0.125	H-atom parameters constrained
*S* = 1.02	*w* = 1/[σ^2^(*F*_o_^2^) + (0.0634*P*)^2^] where *P* = (*F*_o_^2^ + 2*F*_c_^2^)/3
2817 reflections	(Δ/σ)_max_ < 0.001
163 parameters	Δρ_max_ = 0.14 e Å^−^^3^
0 restraints	Δρ_min_ = −0.21 e Å^−^^3^

### Special details

**Table:**

Geometry. All esds (except the esd in the dihedral angle between two l.s. planes) are estimated using the full covariance matrix. The cell esds are taken into account individually in the estimation of esds in distances, angles and torsion angles; correlations between esds in cell parameters are only used when they are defined by crystal symmetry. An approximate (isotropic) treatment of cell esds is used for estimating esds involving l.s. planes.
Refinement. Refinement of *F*^2^ against ALL reflections. The weighted *R*-factor wR and goodness of fit *S* are based on *F*^2^, conventional *R*-factors *R* are based on *F*, with *F* set to zero for negative *F*^2^. The threshold expression of *F*^2^ > σ(*F*^2^) is used only for calculating *R*-factors(gt) etc. and is not relevant to the choice of reflections for refinement. *R*-factors based on *F*^2^ are statistically about twice as large as those based on *F*, and *R*- factors based on ALL data will be even larger.

### Fractional atomic coordinates and isotropic or equivalent isotropic displacement parameters (Å^2^)

**Table:**

	*x*	*y*	*z*	*U*_iso_*/*U*_eq_	
O1	1.0767 (2)	0.57962 (7)	0.09248 (8)	0.0495 (3)	
C8	0.6699 (3)	0.51698 (9)	0.25693 (11)	0.0352 (3)	
C2	0.8660 (3)	0.57392 (9)	0.24134 (11)	0.0340 (3)	
C10	0.7471 (3)	0.70670 (10)	0.39972 (11)	0.0393 (4)	
H10A	0.6198	0.7051	0.3439	0.047*	
N2	0.9971 (2)	0.63123 (8)	0.29290 (9)	0.0400 (3)	
O2	0.9021 (2)	0.79318 (8)	0.66799 (7)	0.0508 (3)	
H2B	0.8087	0.8338	0.6566	0.076*	
C9	0.9565 (3)	0.66564 (10)	0.39018 (11)	0.0353 (3)	
C6	0.4499 (3)	0.40004 (11)	0.15667 (13)	0.0475 (4)	
H6A	0.4190	0.3651	0.0969	0.057*	
N1	0.7719 (2)	0.48427 (9)	0.09663 (9)	0.0444 (4)	
H1A	0.7677	0.4596	0.0365	0.053*	
C12	0.9123 (3)	0.75150 (10)	0.57572 (10)	0.0355 (3)	
C4	0.3698 (3)	0.44199 (11)	0.32725 (13)	0.0486 (4)	
H4A	0.2835	0.4340	0.3808	0.058*	
C3	0.5427 (3)	0.50578 (11)	0.33753 (12)	0.0429 (4)	
H3A	0.5728	0.5405	0.3975	0.051*	
C7	0.6211 (3)	0.46330 (9)	0.16765 (11)	0.0375 (4)	
C1	0.9229 (3)	0.54782 (10)	0.13512 (11)	0.0388 (4)	
C11	0.7262 (3)	0.74994 (10)	0.49164 (11)	0.0373 (4)	
H11A	0.5860	0.7781	0.4968	0.045*	
C5	0.3247 (3)	0.39038 (11)	0.23869 (14)	0.0513 (4)	
H5A	0.2078	0.3481	0.2335	0.062*	
C13	1.1198 (3)	0.70905 (11)	0.56766 (12)	0.0475 (4)	
H13A	1.2441	0.7082	0.6248	0.057*	
C14	1.1421 (3)	0.66800 (12)	0.47483 (12)	0.0473 (4)	
H14A	1.2841	0.6415	0.4691	0.057*	

### Atomic displacement parameters (Å^2^)

**Table:**

	*U*^11^	*U*^22^	*U*^33^	*U*^12^	*U*^13^	*U*^23^
O1	0.0656 (8)	0.0479 (7)	0.0418 (6)	−0.0122 (6)	0.0279 (6)	−0.0074 (5)
C8	0.0407 (9)	0.0316 (8)	0.0344 (7)	0.0034 (6)	0.0096 (6)	0.0006 (6)
C2	0.0396 (9)	0.0333 (8)	0.0311 (7)	0.0018 (6)	0.0118 (6)	−0.0011 (6)
C10	0.0357 (9)	0.0460 (9)	0.0333 (8)	0.0007 (7)	−0.0015 (6)	−0.0071 (6)
N2	0.0444 (8)	0.0408 (7)	0.0370 (7)	−0.0015 (6)	0.0132 (6)	−0.0065 (6)
O2	0.0590 (8)	0.0577 (7)	0.0318 (6)	0.0143 (6)	−0.0024 (5)	−0.0126 (5)
C9	0.0372 (8)	0.0367 (8)	0.0329 (7)	−0.0032 (6)	0.0088 (6)	−0.0056 (6)
C6	0.0539 (11)	0.0398 (9)	0.0491 (9)	−0.0052 (8)	0.0101 (8)	−0.0073 (7)
N1	0.0591 (9)	0.0440 (8)	0.0339 (7)	−0.0101 (7)	0.0185 (6)	−0.0116 (6)
C12	0.0413 (9)	0.0357 (8)	0.0279 (7)	0.0013 (6)	0.0023 (6)	−0.0033 (6)
C4	0.0496 (11)	0.0488 (10)	0.0525 (10)	−0.0006 (8)	0.0228 (8)	0.0083 (8)
C3	0.0518 (10)	0.0416 (9)	0.0386 (8)	0.0025 (7)	0.0174 (7)	−0.0012 (7)
C7	0.0439 (9)	0.0342 (8)	0.0360 (8)	0.0011 (7)	0.0116 (7)	−0.0002 (6)
C1	0.0485 (10)	0.0371 (8)	0.0334 (7)	−0.0006 (7)	0.0140 (7)	−0.0012 (6)
C11	0.0339 (8)	0.0399 (8)	0.0375 (8)	0.0037 (7)	0.0047 (6)	−0.0065 (7)
C5	0.0490 (11)	0.0429 (10)	0.0636 (11)	−0.0070 (8)	0.0148 (9)	0.0040 (8)
C13	0.0405 (10)	0.0587 (11)	0.0384 (8)	0.0079 (8)	−0.0061 (7)	−0.0083 (8)
C14	0.0347 (9)	0.0562 (10)	0.0504 (9)	0.0061 (7)	0.0061 (7)	−0.0123 (8)

### Geometric parameters (Å, °)

**Table:**

O1—C1	1.2271 (18)	C6—H6A	0.9300
C8—C3	1.388 (2)	N1—C1	1.343 (2)
C8—C7	1.403 (2)	N1—C7	1.4103 (18)
C8—C2	1.472 (2)	N1—H1A	0.8600
C2—N2	1.2682 (19)	C12—C11	1.380 (2)
C2—C1	1.5197 (19)	C12—C13	1.383 (2)
C10—C11	1.3835 (19)	C4—C3	1.388 (2)
C10—C9	1.388 (2)	C4—C5	1.376 (2)
C10—H10A	0.9300	C4—H4A	0.9300
N2—C9	1.4199 (17)	C3—H3A	0.9300
O2—C12	1.3624 (16)	C11—H11A	0.9300
O2—H2B	0.8200	C5—H5A	0.9300
C9—C14	1.383 (2)	C13—C14	1.380 (2)
C6—C7	1.375 (2)	C13—H13A	0.9300
C6—C5	1.393 (2)	C14—H14A	0.9300
			
C3—C8—C7	119.14 (14)	C3—C4—H4A	119.6
C3—C8—C2	134.40 (14)	C5—C4—H4A	119.6
C7—C8—C2	106.41 (12)	C4—C3—C8	118.99 (15)
N2—C2—C8	137.84 (13)	C4—C3—H3A	120.5
N2—C2—C1	116.76 (13)	C8—C3—H3A	120.5
C8—C2—C1	105.25 (12)	C6—C7—C8	122.30 (14)
C11—C10—C9	120.48 (14)	C6—C7—N1	127.65 (14)
C11—C10—H10A	119.8	C8—C7—N1	110.06 (13)
C9—C10—H10A	119.8	O1—C1—N1	126.69 (13)
C2—N2—C9	122.96 (13)	O1—C1—C2	126.23 (14)
C12—O2—H2B	109.5	N1—C1—C2	107.08 (12)
C14—C9—C10	118.60 (13)	C12—C11—C10	120.35 (14)
C14—C9—N2	118.64 (14)	C12—C11—H11A	119.8
C10—C9—N2	122.26 (13)	C10—C11—H11A	119.8
C7—C6—C5	117.31 (15)	C6—C5—C4	121.52 (16)
C7—C6—H6A	121.3	C6—C5—H5A	119.2
C5—C6—H6A	121.3	C4—C5—H5A	119.2
C1—N1—C7	111.18 (12)	C12—C13—C14	119.96 (15)
C1—N1—H1A	124.4	C12—C13—H13A	120.0
C7—N1—H1A	124.4	C14—C13—H13A	120.0
C11—C12—C13	119.48 (13)	C9—C14—C13	121.07 (15)
C11—C12—O2	122.94 (13)	C9—C14—H14A	119.5
C13—C12—O2	117.57 (13)	C13—C14—H14A	119.5
C3—C4—C5	120.74 (15)		

### Hydrogen-bond geometry (Å, °)

**Table:**

*D*—H···*A*	*D*—H	H···*A*	*D*···*A*	*D*—H···*A*
O2—H2B···O1^i^	0.82	1.96	2.7628 (17)	165
N1—H1A···O1^ii^	0.86	2.12	2.9071 (16)	153
C10—H10A···O2^iii^	0.93	2.38	3.275 (2)	160
C11—H11A···O1^i^	0.93	2.52	3.117 (2)	122

Symmetry codes: (i) *x*−1/2, −*y*+3/2, *z*+1/2; (ii) −*x*+2, −*y*+1, −*z*; (iii) *x*−1/2, −*y*+3/2, *z*−1/2.
